# Prion and Prion-Like Protein Strains: Deciphering the Molecular Basis of Heterogeneity in Neurodegeneration

**DOI:** 10.3390/v11030261

**Published:** 2019-03-14

**Authors:** Carlo Scialò, Elena De Cecco, Paolo Manganotti, Giuseppe Legname

**Affiliations:** 1 Laboratory of Prion Biology, Department of Neuroscience, Scuola Internazionale Superiore di Studi Avanzati (SISSA), 34136 Trieste, Italy; edececco@sissa.it (E.D.C.); legname@sissa.it (G.L.); 2 Clinical Unit of Neurology, Department of Medicine, Surgery and Health Sciences, University Hospital and Health Services of Trieste, University of Trieste, 34149 Trieste, Italy; pmanganotti@units.it; 3 ELETTRA Sincrotrone Trieste S.C.p.A, Basovizza, 34149 Trieste, Italy

**Keywords:** prion, prion-like proteins, strains, neurodegeneration

## Abstract

Increasing evidence suggests that neurodegenerative disorders share a common pathogenic feature: the presence of deposits of misfolded proteins with altered physicochemical properties in the Central Nervous System. Despite a lack of infectivity, experimental data show that the replication and propagation of neurodegenerative disease-related proteins including amyloid-β (Aβ), tau, α-synuclein and the transactive response DNA-binding protein of 43 kDa (TDP-43) share a similar pathological mechanism with prions. These observations have led to the terminology of “prion-like” to distinguish between conditions with noninfectious characteristics but similarities with the prion replication and propagation process. Prions are considered to adapt their conformation to changes in the context of the environment of replication. This process is known as either prion selection or adaptation, where a distinct conformer present in the initial prion population with higher propensity to propagate in the new environment is able to prevail over the others during the replication process. In the last years, many studies have shown that prion-like proteins share not only the prion replication paradigm but also the specific ability to aggregate in different conformations, i.e., strains, with relevant clinical, diagnostic and therapeutic implications. This review focuses on the molecular basis of the strain phenomenon in prion and prion-like proteins.

## 1. Introduction

Neurodegenerative diseases (NDDs) are characterized by the progressive dysfunction and loss of specific neurons leading to the irreversible damage of functional circuitry, ultimately defining their clinical presentations. NDDs include Alzheimer’s disease (AD), Parkinson’s disease (PD), amyotrophic lateral sclerosis (ALS), frontotemporal dementia (FTD), prion diseases or Transmissible Spongiform Encephalopathies (TSEs) and many others. Although they differ in terms of clinical and neuropathological features, increasing evidence suggests that these disorders share a common pathogenic feature: the presence of deposits of misfolded proteins with altered physicochemical properties in the Central Nervous System (CNS) [1]. Thus, a classification of NDDs is based according to the predominant protein that is deposited in the brain, leading to the definition of proteinopathies or conformational diseases [2]. The main protein component of these pathological deposits may be unique for each disorder, e.g., α-synuclein in Parkinson’s disease (PD), while in other conditions more than one misfolded protein can be involved [1]. Extracellular deposits comprise aggregates with an immunoreactivity for amyloid-β (Aβ) or prion protein (PrP), while proteins found in intracellular deposits include tau, α-synuclein and TAR DNA-binding protein 43 (TDP-43). These proteins associate with sporadic and inherited forms, while in the case of prion protein, an infectious route of transmission is also possible.

**Figure 1:**
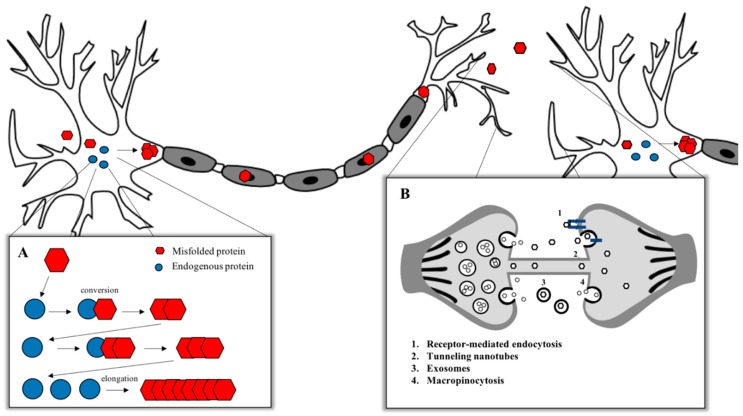
A schematic illustration of the prion-like characteristics of aggregated proteins: (**A**) Once they have entered healthy neurons, amyloids (red hexagons) interact with their native counterparts (blue circles) and act as a template to seed their misfolding. The process is self-amplifying and leads to the formation of long fibrillar aggregates which are transported along the axons toward synaptic terminals. (**B**) The aggregates spread to neighbouring cells via multiple mechanisms, many of which require an active regulation by the cell itself. (1) Endocytosis: Prion-like proteins (synuclein) exploit membrane receptors (LAG3 [32] and prion protein [33]) or clathrin-dependent endocytosis (tau) to enter the cell cytoplasm. (2) Tunneling nanotubes (TNTs): TNTs are involved in the spreading of tau, prions, synuclein and Aβ [34]. (3) Exosomes: Exosomes have been found to be implicated in the spreading of pathological protein aggregates [35]. (4) Macropinocytosis: The tau protein is transferred through macropinocytosis mediated by heparin sulfate proteoglycans [36].

To date, prion diseases are the unique neurodegenerative disorders showing an infectious transmissible protein species capable of recapitulating a clinical disease [3,4] with no clear evidence to the naturally occurring human-to-human transmission of other neurodegenerative conditions [5,6]. Despite this observed lack of infectivity, the high degree of misfolded protein deposition and experimental evidence showing the replication and propagation of various other neurodegeneration related proteins—including Aβ, tau, α-synuclein and TDP-43—suggests that these neurodegenerative diseases share a similar pathological mechanism with prions. These observations have led to the terminology of “prion-like” to distinguish between conditions with noninfectious characteristics and to compare similarities with the prion replication and propagation process [7] (Figure 1). The canonical model for the aggregation of misfolded proteins is based on the prion paradigm [8,9]. According to the “protein-only” hypothesis formulated in 1980 by Stanley B. Prusiner, which coined the term “prion” (proteinaceous infectious particle) [10], the critical pathological step in prion disorders is the conversion of the normal cellular prion protein (PrP^C^) into a β-sheet-enriched pathological conformer PrP-scrapie (PrP^Sc^). The term scrapie derives from the name of a TSE which affects sheep and goats. Even if PrP^Sc^ possesses the same amino acidic sequence of PrP^C^, it acquires specific features that differentiate it from its normal counterpart, like a partial resistance to digestion with proteinase K (PK), an insolubility in nonionic detergents and the enrichment in β-sheet content. PrP^Sc^ is able to induce other PrP^C^ molecules to misfold and aggregate into small oligomers, protofibrils and amyloid fibrils, leading to the pathological process [8,11].

Misfolded protein assemblies in NDDs have been shown to act as seeds of aggregation that can sequester their native isoforms and convert into pathological molecules, thereby growing in size. The term “seed” indicates the smallest amount of a misfolded protein able to template the pathological conversion onto native molecules. The subsequent fragmentation of the aggregates and the repetition of the cycle leads to the amplification of the pathological state within one cell, as well as through the nervous system via the release of seeds to the extracellular space, via the uptake by the neighboring cells and via the repetition of the propagation cycle [2]. According to this concept, a misfolded protein is considered to display prion-like properties if it possesses seeding properties, if it is able to spread and propagate, if it can form structurally unique strains and, finally, if the protein aggregates induce neurotoxicity [4]. Among all the peculiar features of prions, one of the most intriguing is the prion strain phenomenon. Animals affected by prion diseases may develop different pathologies with unique clinical and biochemical outcomes that can be maintained through passages in animals. Prions, thus, are not homogeneous isomorphic particles but rather comprise a mixed population of PrP assemblies that share the ability to structurally convert other PrP molecules [9].

**Figure 2:**
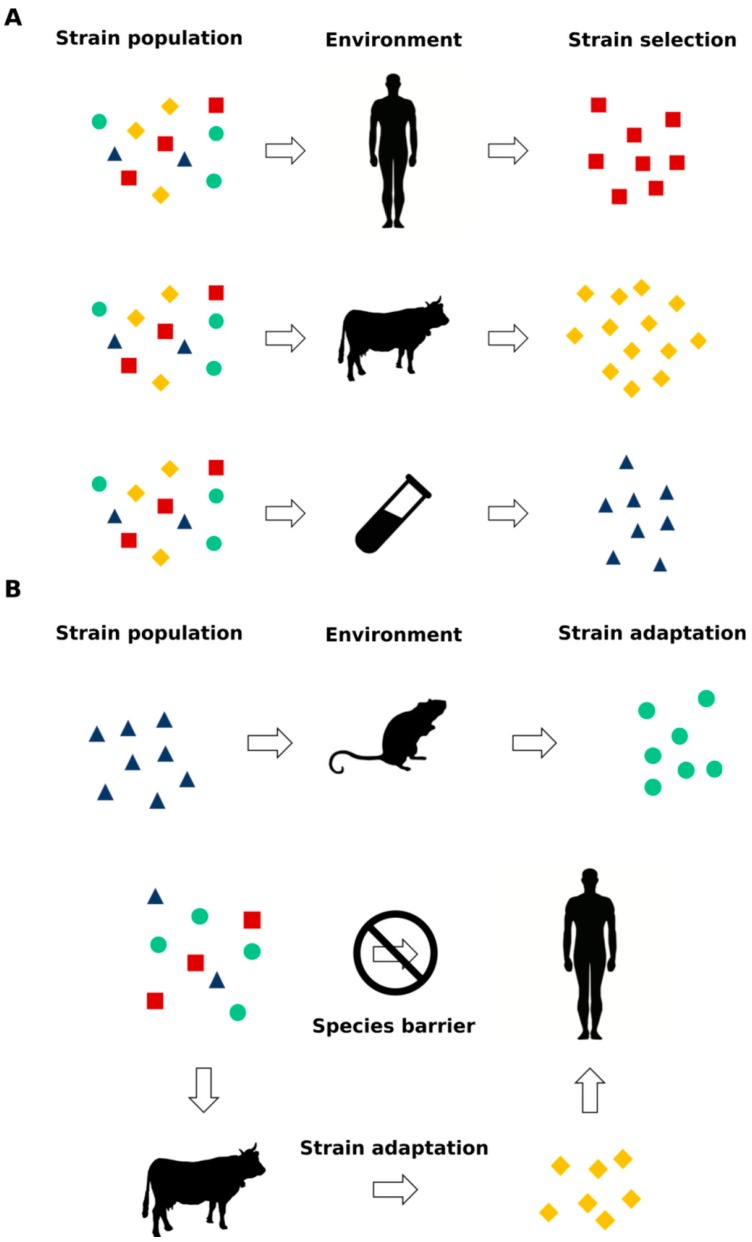
The prion and prion-like strain selection or adaptation: (**A**) According to the replication environment, one prion or prion-like species (i.e., red square in human, yellow diamond in cattle and blue triangle in vitro) present in the initial prion strain population can prevail on the others and be selectively amplified. This phenomenon could manifest in vivo in different animal species, probably influenced by differences in the PrP^C^ sequence of the host, or in vitro according to different experimental settings. (**B**) The appearance of a new prion or prion-like strain can depend on the mechanism of adaptation. In this scenario, one conformer already present in the initial strain population can change its conformation, adapting to the host environment (e.g., blue triangle transforms into yellow diamond). This phenomenon could also explain how prions escape the species barrier. These two processes are not mutually exclusive.

Prions are now considered to be able to adapt their conformation to changes in the context of the environment of replication [12,13,14], and studies on synthetic prions [15,16] revealed that this phenomenon is replicated not only in animal models but also in vitro in cells. This process is known as either prion selection or adaptation, where a distinct PrP^Sc^ conformer present in the initial prion population with a higher propensity to propagate in the new environment is able to prevail over the others during the replication process [12] (Figure 2). Increasing evidence is suggesting that prion-like proteins such as Aβ, α-synuclein, tau and TDP-43 share not only the prion replication paradigm but also this specific ability to aggregate in different conformations, i.e., strains [17,18,19,20,21], with relevant clinical, diagnostic and therapeutic implications. 

Familial AD patients with mutations in the Aβ precursor protein (*APP*) gene tested negative for the amyloid-specific positron emission tomography (PET) probe, Pittsburgh compound B (PiB), which is commonly used to detect Aβ deposits in sporadic AD (sAD) cases [22], despite showing a severe cerebral amyloid burden in postmortem analysis [23], suggesting the presence of a structural difference between Aβ deposits in these subjects when compared to sporadic patients [24,25]. Several studies have shown the presence of different Aβ conformers in amyloid deposits not only in subjects harboring an *APP* gene mutation but also in patients with different AD clinical subtypes [19,20,21]. Similarly, it has been showed that tau strains derived from distinct human tauopathies are propagated in vivo through intracranial injections into wild-type mice and transgenic mouse models of AD; these pathologies closely recapitulate distinct features of human AD and its associated tauopathies [18]. Moreover, it is currently hypothesized that under specific disease conditions, endogenous α-synuclein assemblies aberrantly fold to form prion strains [26,27,28] and that, after exogenous administration, pathogenic α-synuclein inflicts pathology by amplifying in a permissive environment via different routes [27,29,30,31]. A deep understanding of the molecular basis leading to the strain formation and propagation could be of utmost relevance to carefully approach, to classify and, ultimately and hopefully, to cure different entities belonging to the same neurodegenerative disorder. In this context, these diseases could be better addressed as “conformational disorders” instead of the more generic definition of proteinopathies. Given the relevance of this topic, this review will highlight the evidence pointing to the existence of prion and prion-like protein strains, starting from the well-established notions about the prion protein and subsequently moving to the emerging data regarding the other prion-like proteins involved in neurodegeneration.

## 2. Prion Protein

### 2.1. The Cellular Prion Protein

The cellular prion protein, PrP^C^, is a glycosylphosphatidylinositol (GPI)-anchored protein of 231 amino acids encoded by the *PRNP* gene located on chromosome 20 in humans and on chromosome 2 in mice [37,38,39,40,41]. Its sequence can be divided into two structurally well-defined regions: a long, N-terminal flexible tail containing series of four or five repeats of eight amino acids (PHGGGWGQ) and a globular C-terminal domain containing 3 α-helices and 3 β-strands, 2 of which flank the first α-helix. As a typical cell-surface glycoprotein, the pre-pro-protein is translocated to the endoplasmic reticulum (ER) where it is subjected to several posttranslational modifications including the N-linked glycosylation at residues N181 and N197 in humans, the formation of a single disulfide bond at position C179 and C214, the cleavage of the C-terminal signal peptide and the subsequent attachment of the glycosylphosphatidyl inositol (GPI) anchor at position 231 [42]. PrP^C^ is widely expressed in the CNS during early development and in adult neurons and glial cells. In the adult brain, maximal *PRNP* mRNA expression is observed in the neocortex and cerebellum. In addition to the nervous system, the mammalian expression of PrP^C^ has been reported in several tissues including lymphoid organs and the heart [40,43,44] and at lower levels in the kidney and liver [44,45]. Even if the PrP^C^ physiological role is far from being completely understood, several putative functions have been suggested for PrP^C^, including neuritogenesis, neuronal homeostasis, cell signaling, cell adhesion and a protective role against stress [46]. In humans, prion disorders or TSEs include idiopathic forms such as sporadic Creutzfeldt-Jakob disease (sCJD), sporadic fatal insomnia (sFI) and the variably proteinase sensitive prionopathies (VPSPr). Familial CJD (fCJD), Gerstmann–Sträussler–Scheinker disease (GSS), fatal familial insomnia (FFI) and prion protein cerebral amyloid angiopathy (PrP-CAA) are genetic forms of human TSEs. The acquired forms are transmitted from human to human as iatrogenic CJD (iCJD) and Kuru or from cattle to human as variant CJD (vCJD) [47]. In animals, TSEs include scrapie in sheep and goats, bovine spongiform encephalopathy (BSE) in cattle and chronic wasting disease (CWD) in cervids [48].

### 2.2. Molecular Basis of Prion Strains

Stanley B. Prusiner elegantly and unambiguously showed that the scrapie infectious agent was a proteinaceous particle devoid of nucleic acids. Pattison and Millson [49] had already observed the existence of different prion strains in goats. In their reports, they described two different clinical phenotypes in goats affected by the same batch of the scrapie agent. The authors called these phenotypes “scratching” and “nervous” syndromes according to the main clinical manifestations of the animals. Even if they were not aware of the nature of the infectious agent, they clearly recognized that the two syndromes were reproducible by the intracerebral inoculation of animals, suggesting that certain "strains" of the scrapie agent produced the nervous syndrome, while others produced the scratching one [49]. In microbiology, a strain is defined as an isolate or group of isolates that can be distinguished from other isolates of the same genus and species by phenotypic characteristics or genotypic characteristics or both [50]. Since the nature of the scrapie infectious agent was unknown, the description of the scrapie strains initially seemed to be an indirect proof for the existence of a different genetic information encoded by a specific scrapie-causing agent, thus conflicting with the protein-only hypothesis. However, using different approaches, including Fourier transform infrared spectroscopy (FTIR), atomic force microscopy (AFM) and circular dichroism (CD), it has been shown that differences in prion strains lie in the different conformations of PrP^Sc^ [51,52,53,54,55,56]. Prion strains can be classified according to different parameters, which can be divided in two main groups, mutually related one to the other: in vivo characteristics [57,58,59] and biochemical properties of the infectious protein [52,60,61,62,63]. The most commonly used in vivo criteria for the classification of a given prion strain are the incubation period, which is defined as the time elapsed between an experimental inoculation of a given dose and the clinical onset of disease; the survival time; and the main clinical signs (e.g., rough coat, ataxia and loss of weight) [13]. Other parameters for the in vivo strain classification are neuropathological differences in terms of the preferential distribution of PrP^Sc^ deposition in the CNS (e.g., synaptic, peri-neuronal, plaque-like and amyloid plaques) and the degree of vacuolation in specific brain regions of the affected animal [12,64,65,66,67]. In order to quantify these aspects, a standardized procedure in mice, called lesion profile, for the scoring of vacuolization in a number of gray matter and white matter brain areas (depending on the scoring system used) of the affected animal has been developed [13]. The most relevant biochemical properties of the infectious protein appreciated in different prion strains are the electrophoretic mobility after PK digestion, the glycosylation pattern (i.e., the prevalence of the mono- or di-glycosylated form), the extent of PK resistance and sedimentation, and the resistance to denaturation by a chaotropic agent [13,52,60,61,62,63].

### 2.3. Strain Diversity in Human TSEs

**Figure 3:**
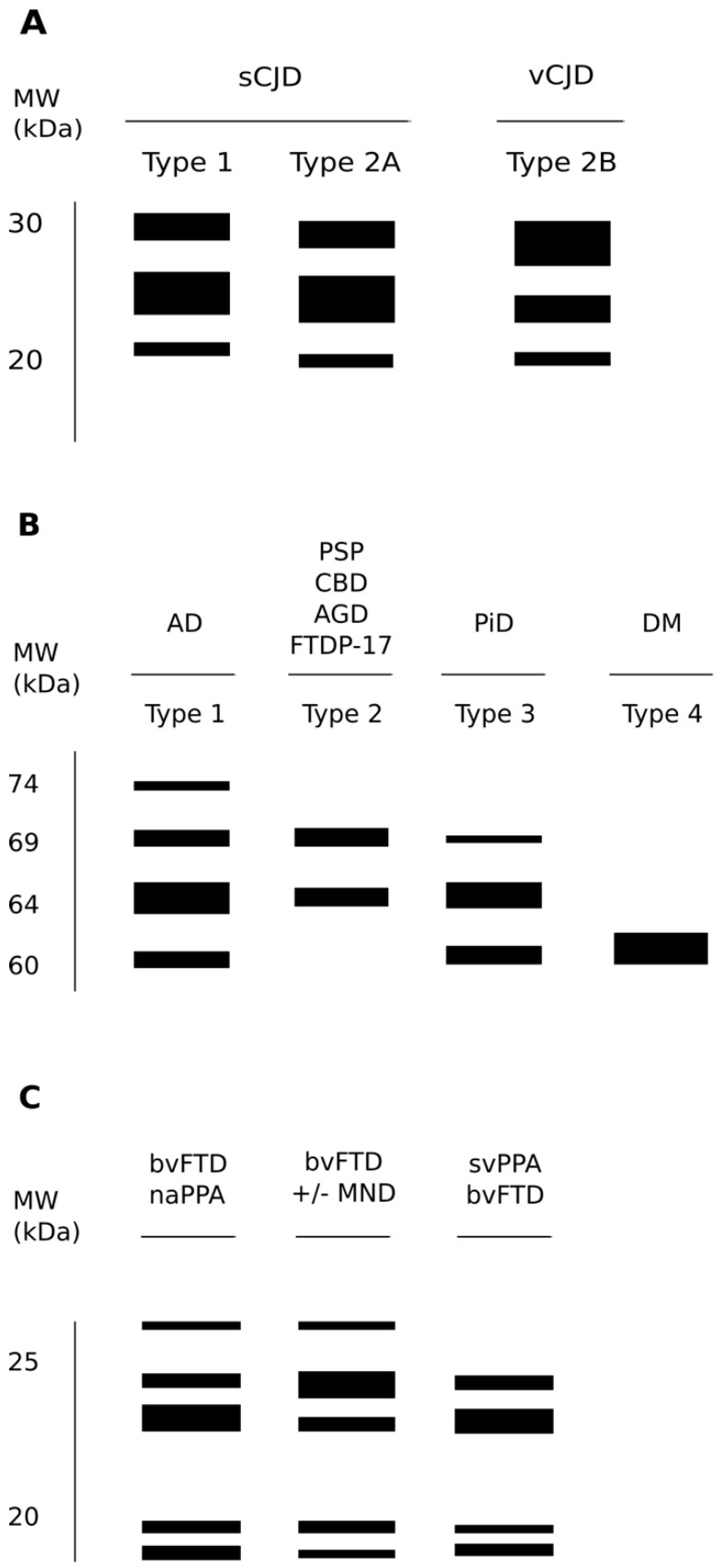
Representative western blot profiles of the prion and prion-like pathological proteins extracted from diseased brains: (**A**) A schematic representation of human PrP^Sc^ types after PK digestion. In the sCJD cases, the PK cleavage of PrP^Sc^ results in type 1 when the unglycosylated PK-resistant fragment presents a molecular mass of 21 kDa and in type 2A when the it has a molecular mass of 19 kDa. In both cases, there is a predominance of the mono-glycosylated band over the others. vCJD presents a different pattern associated with the presence of the unglycosylated PK-resistant fragment of 19 kDa and the predominance of the di-glycosylated band in the glycoform ratio (type 2B). (**B**) The four different electrophoretic patterns of pathological tau proteins are composed of bands at 60, 64, 69 and 74 kDa, which correspond to the pathological tau that are found in aggregates in different tauopathies (listed on the top of each pattern). (**C**) The representative immunoblots with the phosphorylated TDP-43 specific antibody, pS409/410. FTLD-TDP type B pathology shows three major bands at 23, 24 and 26 kDa and two minor bands at 18 and 19 kDa, with the predominance of the 24 kDa band. Type C pathology shows two major bands at 23 and 24 kDa and two minor bands at 18 and 19 kDa with the 23 kDa band being the most represented. Type A pathology is not distinctive but intermediate between the other two. sCJD: sporadic Creutzfeldt-Jakob disease; vCJD: variant Creutzfeldt-Jakob disease; AD: Alzheimer’s Disease; PSP: Progressive Supranuclear Palsy; CBD: Corticobasal Degeneration; AGD: Argirophilic Grain Disease; FTLD-17: Frontotemporal Dementia linked to chromosome 17; PiD: Pick’s Disease; DM: myotonic dystrophy; bvFTD: behavioural variant of Frontotemporal Dementia; naPPA: nonfluent/agrammatic primary progressive aphasia; MND: motor neuron disorder; svPPA: semantic variant primary progressive aphasia.

PrP^Sc^ strains are considered to be at the basis of the wide variability of human TSEs in terms of clinical signs, incubation periods, neuropathological manifestations and biochemical properties of the deposited PrP^Sc^. Since human prion disorders are characterized by a plethora of possible manifestations that appear with more or less intensity depending on the strain of the agent causing the disorder, clinical signs represent a powerful guide to discriminate between different entities. For example, sCJD patients usually present with cognitive decline, ataxia or visual disturbance either alone or in combination [68,69], while vCJD cases are caused by the transmission of BSE to humans; often psychiatric symptoms prevail, usually preceding neurological signs [70]. In other genetic forms of human TSEs, like FFI, subjects experience the inability to initiate and maintain sleep, with frequent arousals and enacted dreams associated with autonomic alterations [71]. Intriguingly, to complicate even more the variety of clinical manifestations linked to prion agents, different groups reported familial cases of prion disorders linked to the genetic mutations in the *PRNP* gene and characterized by severe chronic diarrhea and autonomic alterations in the apparent absence of the involvement of the CNS [72].The different nature of the biochemical features of PrP^Sc^ in several subtypes of sCJD and in the sporadic form of fatal insomnia (sFI) led to a classification system based on two distinct patterns of electrophoretic mobility of the unglycosylated protease-resistant fragment of PrP^Sc^ (type 1 and 2) and on the associated genetic polymorphism at codon 129 of the *PRNP* gene (methionine or valine; MM, MV and VV) [69]. Furthermore, Western blot (WB) patterns depend on the PK cleavage of PrP^Sc^ at different sites resulting in type 1 when the unglycosylated PK-resistant fragment presents a molecular mass of 21 kDa and in type 2 when it has a molecular mass of 19 kDa (Figure 3A) [69]. Interestingly, this classification shows a strong correlation between the clinical phenotype, the neuropathological features of PrP^Sc^ deposition and the biochemical and structural properties of the specific strain of the deposited PrP^Sc^, thus giving a strain-related explanation of the extreme clinical heterogeneity of sCJD. Further evidence providing a correlation between the specific pathologic phenotypes and the structural and biochemical features of PrP^Sc^ in human prion disorders derives from the demonstration that what distinguishes the genetic of FFI to a specific familial form of CJD (fCJD^178^) is the genotype at the polymorphic codon 129 in the allele of the *PRNP* gene which contains the same pathogenic point mutation at codon 178. In fact, FFI is an autosomal dominant prion disorder linked to a mutation in codon 178 of the *PRNP* gene, resulting in the substitution of aspartic acid with asparagine (D178N); however, the same mutation is also linked to fCJD^178^, but in FFI, the D178N mutation is aligned with methionine at codon 129 (D178N-129M haplotype), while in fCJD^178^, there is valine (D178N-129V haplotype) [73]. This proves that the 129M/V polymorphism associated with a single mutation can determine different phenotypes. Furthermore, WB analyses showed that the PK-resistant PrP^Sc^ has a different electrophoretic mobility in these two disorders, having the unglycosylated band at an electrophoretic mobility of 19 kDa in FFI and of 21 kDa in fCJD^178^ [74]. Further complicating this scenario is the existence of a sporadic form of fatal insomnia (sFI) not related to any mutation in the *PRNP* gene but displays overlapping clinical features and the same PK-resistant PrP^Sc^ electrophoretic mobility pattern of FFI, thus suggesting that a similar prion strain is responsible for these two disorders [69].

### 2.4. Species Barrier, Prion Strains Transmission, Selection and Adaptation

Since a strain is defined through its clinical, neuropathological and structural properties, the use of animal models has been instrumental to further characterize and define prions. Among all experimental animal models, mice have discriminated more than 20 different prion strains so far [13]. Strain isolation is usually obtained through the inoculation of scrapie-infected material from goat and sheep, BSE-derived material from cattle or human sources of PrP^Sc^ from the brains of deceased subjects with sCJD or GSS [65,66,75,76,77,78]. Usually, in order to obtain a stabilized strain, several passages of the same source in one species with a constant background are needed. Examples of mouse-adapted scrapie prion strains are RML, ME7, 139A and 79A, while mouse strains generated by the inoculation of BSE and sCJD prions are the 301C and Fukuoka, respectively [79,80,81]. Interestingly, the agent responsible for prion disorders seems to be able to infect some animal species and not others. This phenomenon, known as “species barrier” depends mainly on genetic variability and on sequence difference of PrP^C^ in the host animal and explains the prolongation in the incubation period observed [82,83]. When the PrP^C^ host structure is completely resistant to prion infection, the species barrier is considered absolute. One example of an absolute species barrier is the rabbit, which has been classified as TSE-resistant for four decades, until 2012, when this absolute barrier was overcome with the use of in vitro prion amplification using the technique Protein Misfolding Cyclic Amplification (PMCA) (see next Section), demonstrating the transmissibility of the amplified product in 2 out of 10 rabbits, which can no longer be considered as completely resistant to prions [84].

The only known prion agent capable of transmission between animals and humans is BSE, and it is currently accepted that the consumption of BSE-infected products is the cause of vCJD [85,86]. Transmission studies had a crucial role in establishing a causal link between BSE and vCJD. BSE and vCJD had similar incubation periods and lesion profiles in infected mice brains, which were distinct from those of both scrapie and sCJD [87]. vCJD PrP^Sc^ displays peculiar biochemical properties characterized by the presence of the unglycosylated PK-resistant fragment of 19 kDa and the predominance of the di-glycosylated band [88]. Clinically, vCJD differs from all other human prion disorders, being characterized by early and persistent psychiatric symptoms and followed, after some months, by neurological signs [89]. Intriguingly, until 2017 [90], all probable/definite vCJD-reported cases were homozygous for methionine (MM) at codon 129 of the *PRNP* gene, thus, suggesting an absolute barrier displayed by a PrP^C^ sequence associated with MV or VV genotype (i.e., no clinically affected subjects were MV or VV carriers). In 2017, the first case of an affected subject harboring the MV genotype which displayed typical vCJD neuropathological and biological features was described [90]. Furthermore, a biochemical analysis of the spleen of asymptomatic MV subjects which received a blood donation from vCJD donors [91,92] showed the presence of pathological prion protein deposition, which, in one case, displayed also infectivity when injected in mice [91]. These studies demonstrated that spleen tissue from an asymptomatic *PRNP* MV genotype subject can propagate the vCJD disease agent and that the infectious protein can be present in the spleen without CNS involvement.

Altogether, these data created surveillance concerns regarding the presence in the population of MV subjects with subclinical vCJD. Furthermore, an elegant study conducted on a large cohort of nonhuman primates showed that vCJD infectious agent(s) contained in soluble or insoluble fractions of vCJD blood donors is/are able to replicate in macaques generating typical and nonconventional vCJD phenotypes, the latter characterized by the presence of PK-sensitive PrP^Sc^ deposition and atypical clinical features mainly involving the spinal cord [93]. These results clearly open several questions regarding the design of appropriate vCJD surveillance protocols. The appearance of new prion strains after infection with the same source of PrP^Sc^ suggests that prions are able to modify their conformation to changes in the context of replication: This ability may derive from the adaptation of the prion pathological structure to a new environment or from the selection of a single prion species, which prevails under specific replication conditions (Figure 2) [12,13]. One clear example of this phenomenon is the isolation of two different prion strains after the inoculation of a Syrian hamster with the transmissible mink encephalopathy (TME) agent [57]. This interspecies transmission of prions presented the expected behavior of the species barrier phenomenon: a long incubation period after the first infection followed by a shorter incubation periods after several passages. The incubation intervals became stable in two groups of animals with different clinical manifestations: the first one with an incubation period of approx. 150 days postinoculation (dpi) presented lethargy (DY), while a shorter incubation period strain (approx. 60 dpi) presented hyperactivity (HY) [57]. A biochemical and histological analysis of the CNS showed that the PrP^Sc^ of the two groups possessed different electrophoretic mobilities, a distinct resistance to PK digestion and stability against guanidine hydrochloride and induced different neuropathological alterations [51,60,94].

These observations suggest two possible hypothesis: in the first one, the infectious strain exists as a single structural species and the appearance of a new one derives from a sort of mutation that occurred during the replication process (i.e., adaptation); in the second one, the infectious strain is composed by a population of different conformers with a dominant type of PrP^Sc^ preferentially replicating in a specific host, and in another one, a different subtype prevails (i.e., selection) according to the new replication environment (Figure 2). Although this phenomenon is still not completely understood, the DY and HY example provided direct evidence of the ability of prions to undergo the process of adaptation or selection according to the environment of replication (mink vs. hamster CNS). Furthermore, it has been shown that brain-derived prion strains replicating in cell cultures can evolve following a Darwinian model when exposed to a drug-dependent selective pressure [95]. The high impact of the prion strain phenomenon on therapeutic drug efficacy and, thus, development was confirmed in another elegant study which demonstrated that prion strains can acquire resistance upon exposure to a specific anti-prion compound (called IND24) and that this resistance is lost upon passage in mice not exposed to the molecule [96]. The advent of synthetic prions [15,16] enabled the study of the prion-strain phenomenon in a highly pure and reproducible environment, providing a powerful tool for the study of this process.

### 2.5. Synthetic Prion Strains

As stated before, conversely to PrP^C^, PrP^Sc^ is rich in β-sheet structures and its partial proteolytic digestion leads to the formation of a PK-resistant core, PrP^res^. In most cases, in vitro-generated PrP^res^ failed to induce any prion pathology after inoculation in susceptible animals, suggesting that the acquisition of proteinase resistance was not enough for the propagation of infectivity [97,98,99,100]. Intracerebral inoculation into transgenic mice expressing the mutant PrP (P101L) using a synthetic PrP peptide carrying the homologous P101L pathogenic mutation and forced to refold in β-sheet structures induced prion disease after about 200 dpi [101,102]. This disease was not transmissible to wild-type animals. Pathological alterations were not observed when the injection was performed with peptides harboring the same sequence but lacking the β-sheet structures [103]. In another set of experiments, recombinant PrP (recPrP) was induced to misfold and acquired two different conformations: β-oligomers (in the presence of acidic pH and urea) or fibrillar structures (neutral pH and low concentration of urea) [98]. This reaction was also performed in the presence (seeded) or in the absence (unseeded) of recPrP-preformed amyloid aggregates, and then, the seeded and unseeded products where assessed for infectivity by intracerebral inoculation in transgenic mice (Tg9949) overexpressing the N-terminally truncated mouse PrP (residues 89–230). In both cases, the animals succumbed to prion disease but with different neuropathological alterations [16]. Specifically, animals treated with the seeded product exhibited a shorter incubation time associated with higher PrP^res^ deposition in the CNS. The isolate collected from those animals was called MoSP1 and was passaged to wild-type mice which were efficiently infected and showed the presence of PrP^res^ deposition in their brains [16]. These results provided relevant information about the relationship between the stability of a specific prion isolate and the length of incubation time after inoculation in susceptible animals, which showed to be directly correlated [15,104].

In order to better characterize the relationship between conformation, stability and infectivity, several laboratories started to produce synthetic prions showing that not all the obtained amyloids were infectious and those which were infectious induced neuropathological changes characterized by a unique pattern of PrP deposition, revealing that each inoculum was a unique isolate and that the properties of this isolate relied on its specific sequence and structure [46]. Recently, one group reported the synthesis of another human prion strain derived from the in vitro aggregation of human recombinant non-glycosylated prion protein seeded with sCJD MM1 prions, using ganglioside GM1 as a cofactor [105]. These synthetic human prions were infectious to transgenic mice expressing non-glycosylated human prion proteins, causing neurologic dysfunction after 459 and 224 days in the first and second passage, respectively. These results confirmed that prion infectivity, host range and the ability to target specific mice brain structures could be related to changes in the structural organization of critical domains, some linked to posttranslational modifications of the pathogenic prion protein (PrP^Sc^). As mentioned above, synthetic prion strains offered as useful tools to decipher the prion selection and adaptation phenomena. When the MoSP1 strain was serially transmitted to wild-type mice, at least two different prion isolates were observed, namely MoSP1(1) and MoSP1(2) [106,107]. Both strains were characterized by two different molecular masses of the PK-resistant core, 21 and 19 kDa, respectively [108].

Another strategy to a generate synthetic prion was based on the PMCA technique [109]. In this case, infectious PrP^Sc^ is mixed with an excess of PrP^C^. Cycles of incubation and sonication are repeated several times. The sonication disaggregates PrP^Sc^ fibrils, thus generating different seeds that can recruit and convert normal PrP^C^. After several rounds of PMCA using recPrP that has never been exposed to any prion, a de novo generation of PrP^res^ was observed. In particular, two types of self-perpetuating PrP^res^ characterized by two different PK-resistant cores of 17 and 14 kDa, respectively, were generated [110]. However, only the PrP^res^ of 17 kDa was highly infectious to mice. PMCA has also been exploited to assess different aspects of prion diseases, including the species barrier and the strain adaptation phenomena [84,111,112,113]. Recently, our group showed the ability of a synthetic mouse prion previously generated [114] to change its biochemical properties when challenged in vivo or in vitro by means of PMCA. In vivo experiments revealed that after the serial transmission passages, it was possible to identify two distinct conformations of PrP^Sc^. One of these conformations was characterized by a prevalence of the di-glycosylated isoform of PrP^Sc^ (PrP^Sc^-D), while the other was characterized by the prevalence of the mono-glycosylated isoform (PrP^Sc^-M). Both abnormal conformations were associated with distinct clinical, biochemical and neuropathological alterations. A PMCA analysis of PrP^Sc^-D and PrP^Sc^-M revealed that, regardless of the inoculum, the amplified product was characterized by a prevalence of the di-glycosylated form of PrP (PrP^Sc^-PMCA) [12]. These data suggest that the synthetic prions can adapt their conformation according to changes of the context of replication.

## 3. Amyloid-β

### 3.1. Amyloid-β Peptide Formation, Alzheimer’s Disease and Its Atypical Variants

Alzheimer’s disease (AD) is characterized by the accumulation of the amyloid-β peptide (Aβ) within the brain, associated with the deposition of hyperphosphorylated and cleaved forms of the microtubule-associated protein tau. Compelling evidence suggests that physiologic generation of the neurotoxic Aβ peptide from the sequential cleavage of the much larger transmembrane protein amyloid precursor protein (APP) is the crucial step in the development of AD [115,116]. The *APP* gene is located on chromosome 21, and the protein product is a member of a family of related proteins that includes the amyloid precursor protein-like APLP1 and APLP2, which are all single-pass transmembrane proteins with a large extracellular domain and are all processed in a similar manner [116]. Despite many roles being suggested for APP [116], its precise physiological function is not known and remains a relevant unanswered issue in deciphering AD pathology.

The complete deletion of APP in mice (and of Aβ formation) produces very little phenotype modifications, thus suggesting that a loss of APP or Aβ function is not deleterious [116]. The N-terminus of Aβ is located in the extracellular domain of APP, 28 amino acids from the transmembrane region, while its C-terminus is located in the transmembrane region. The enzymes responsible for cleavage at the N- and C-termini are called β-secretase, or β-site amyloid precursor protein cleaving enzyme 1 (BACE1), and γ-secretase, respectively. The latter is a multiprotein complex composed of presenilin 1 (PS1) or presenilin 2 (PS2) and other transmembrane proteins [117]. A third enzyme α-secretase cleaves between residues 16 and 17, precluding Aβ formation [115]. In fact, APP can be proteolyzed by α-secretase and then γ-secretase, a process that does not generate Aβ, or can be internalized into clathrin-coated vesicles and then cleaved by β-secretase and γ-secretase. The latter process results in the production of Aβ, which is then released into the extracellular space. Following BACE1 cleavage, the APP C-terminal fragment is cleaved by the γ-secretase complex at one of several sites varying from +40 to +44 to generate Aβ peptides (1–40 and 1–42 being most common) and the APP intracellular domain [116]. There are 32 *APP*, 179 *PSEN1* (presenilin 1 gene locus) and 14 *PSEN2* gene mutations that result in early onset, autosomal dominant, fully penetrant AD. The common thread to all these mutations is that they increase the production of the less soluble and more toxic Aβ42 relative to Aβ40 species [118].

In Down’s syndrome, the overexpression of APP results in Aβ deposition in the brain when individuals are in their late twenties. Neurofibrillary tangles develop later and correlate with the onset of the midlife cognitive decline that is common in these individuals [119]. The risk for developing late-onset AD is related to the presence of the apolipoprotein E (APOE) E4 allele which increases the risk for AD to three-fold in individuals carrying one copy of it and to 15-fold in homozygous subjects [116].

The typical AD dementia syndrome has at its clinical core, an amnestic syndrome of the hippocampal type, followed by deficits in word-finding, spatial cognition, executive functions and neuropsychiatric changes [120]. In addition to its typical presentation, non-amnestic atypical variants of AD have been described [121]. Atypical presentations of AD include posterior cortical atrophy (PCA), logopenic progressive aphasia (LPA) and the frontal variant of AD (fvAD). In PCA, the onset is characterized by early, higher-order visual deficits with a higher deposition of neurofibrillary tangles in the occipital regions than in typical AD. LPA is an atypical language variant defined as a primary phonological loop deficit leading to impaired memory, sentence repetition and comprehension, with sparse spontaneous speech and frequent prolonged word-finding pauses. Greater numbers of neurofibrillary tangles within the frontal lobes are seen in fvAD, resulting in a more severe disease course characterized by early behavioral alterations and executive dysfunction [120]. Recently, some cases of AD presenting with a very fast time course of progression resembling the one of CJD have been described, named rapidly progressive AD (rpAD). It has been shown that these cases do not differ to typical AD patients in terms of neuropathological features, thus suggesting that Aβ variants (i.e., strains) could be implicated in their atypical and faster presentation [122,123]. The correlation between AD phenotype and the presence of different Aβ conformers within the CNS will be discussed more in details later in this review.

### 3.2. Prion-Like Properties of Aβ

After inconclusive attempts of transmission of AD pathology in nonhuman primates [124], one group injected AD brain homogenates intracerebrally into marmosets (*Callithrix jacchus*) and, after an incubation period of approximately 6–7 years, found an increase in senile plaque load with associated argyrophilic dystrophic neurites and cerebral amyloid angiopathy in the brain in the absence of neurofibrillary tangles [125]. Subsequently, after the introduction of the *APP* transgenic mouse model, several groups performed the injection of Aβ-rich brain extracts from AD patients or aged *APP* transgenic mice into *APP* transgenic hosts [126,127,128,129,130,131,132,133,134,135,136] showing that it was possible to recapitulate Aβ deposition (i.e., Aβ plaques and cerebral Aβ angiopathy) even using sub-attomolar amounts of brain-derived Aβ [137]. Furthermore, it was shown that Aβ aggregation in the brain can be triggered by seeds delivered to the peritoneal cavity [138,139] and that stainless-steel wires coated with Aβ-rich brain extract were able to induce plaque formation following implantation in the brain of *APP* transgenic mice [140].

In another work, the kinetics of spontaneous and induced Aβ deposition in living mice has been investigated using bioluminescence imaging (BLI) [141]. The uninoculated biogenic mice Tg (*APP*23:Gfap-luc) showed an increase in the BLI signal (i.e., deposition of fluorescent Aβ) in their brains starting at 416 ± 9 dpi, while mice intracerebrally inoculated with brain homogenates from two aged Tg mouse models, Tg (*APP*23) or Tg (*CRND8*), displayed an increase in the brain BLI signal at 261 ± 8 and 238 ± 12 dpi, respectively. The BLI signal remained low in mice inoculated with aged non-Tg brain homogenate until 333 ± 9 dpi. Interestingly, despite unilateral inoculation, the pathology was bilateral, suggesting the progressive spread of Aβ deposition throughout the brain. The same group performed the injection experiments also with the recombinant synthetic Aβ aggregates. In these experiments, Aβ aggregates were obtained in vitro and then intracerebrally inoculated into young Tg (*APP*23:Gfap-luc) mice giving rise to an increase and an anticipation of the emission of the brain BLI signals [141,142]. Interestingly, pathological investigations showed that synthetic Aβ 40 prions produced amyloid plaques containing both Aβ 40 and Aβ 42 species in the brains of inoculated mice, whereas synthetic Aβ 42 stimulated the formation of smaller, more numerous plaques composed predominantly of Aβ 42 [142]. However, as with PrP, the seeding power of synthetic Aβ was less than the one displayed by Aβ aggregates obtained from diseased brain, thus suggesting the opportunity that cofactors present within the CNS could facilitate Aβ spreading [9]. Recently, Purro et al., analyzed cadaveric pituitary-derived growth hormone (c-hGH) batches used in the past to treat patients that subsequently developed iCJD associated with relevant Aβ deposition, showing that they contain substantial levels of Aβ40, Aβ42 and tau proteins. Furthermore, this material was able to seed the formation of Aβ plaques and cerebral Aβ-amyloid angiopathy in intracerebrally inoculated mice expressing a mutant, humanized amyloid precursor protein [143]. It is important to note that, although these studies suggest that Aβ aggregates behave like prions at the molecular level, there is currently no evidence that AD is infectious in the sense that it is transmissible among humans [144,145].

### 3.3. AD Heterogeneity: Aβ Strains

Given that the large phenotypical heterogeneity of prion disorders can be explained by the presence of different strains of PrP^Sc^, after the discovery of prion-like features of Aβ, increasing interest has been directed towards the study of the structure of Aβ molecules within AD diseased brains and its potential link with AD clinical diversity. The first evidence that Aβ polymorphism may correlate with variations in clinical and pathological features of AD derives from the observation that Aβ40 fibrils with different molecular structures exhibit different levels of toxicity in primary neuronal cell cultures [146]. In another study, fibrils extracted from AD brains seeded the aggregation of synthetic Aβ in vitro obtaining different Aβ descendant species, thus providing indirect evidence for the structural heterogeneity of Aβ among AD brains. The same study also showed the presence of a pool of Aβ conformers in the same brain with a single dominant species among the others [147]. Other proofs of the existence of intersubject, different Aβ conformers derive from studies on rapidly progressive AD (rpAD) brains. Using novel biophysical techniques, as conformation-dependent immunoassay (CDI) and conformational stability assay (CSA), it was shown that even if rpAD cases do not differ from classical cases on a neuropathological basis, they display the presence of Aβ42 fibrils with specific biochemical features, like the size distributions of aggregates and their resistance to chemical denaturation [122,123]. Furthermore, Aβ fibrils of an AD diseased brain with a very high degree of Aβ deposition tested negative when probed with the high-affinity Pittsburgh compound B (PiB), suggesting the presence of a different Aβ conformer presenting a low density of PiB binding sites [148]. This was also the case of familial AD patients with the Arctic (Aβ(E22G)) or Osaka (Aβ(E22Δ)) mutations in the Aβ precursor protein (*APP*) gene, which tested negative for amyloid PET with the PiB compound, which is commonly used to detect Aβ deposits in sporadic AD (sAD) cases [22] despite showing a severe cerebral amyloid burden in a postmortem analysis [24,25].

Recently, three different groups focused their attention on the study of the correlation between AD diversity and the presence of structural Aβ conformers within the same brain of AD subjects or between subjects displaying atypical phenotypic features or harboring a disease-causing *APP* mutation [19,20,21]. In the first of these studies, luminescent conjugated oligothiophenes (LCOs) were used and biochemical analyses were carried out to evaluate the variation and structural properties of amyloid in the plaques of patients with AD from different etiological backgrounds (familial forms, sporadic forms and one single PiB-negative AD case) providing evidence for the existence of heterogeneous Aβ-amyloid structures that cluster across different patients with AD. Furthermore, the Aβ-rich brain extracts from different subtypes of AD transmitted to Tg mice and seeded Aβ deposits correspondingly differentiable using LCOs which mirrored, even if not completely, those present in the donor brain. The authors suggest that this uncomplete correspondence of fibril structures between donors and recipients was probably due to the presence of a mixture of Aβ species in the donor extract which may follow a Darwinian strain selection [20].

In another study, Condello et al. showed that Aβ deposits from fAD (Aβ(E22G)) and fCAA (Aβ(E22Q)) patients could be distinguished from sAD patients based on the shape of the emission spectra of multiple amyloid-staining dyes which are influenced by the conformational Aβ strain. They also inoculated Tg mice expressing only WT Aβ with synthetic Aβ40 fibrils containing fAD-associated mutations, showing that mutant Aβ induced the formation of plaques with reduced intensity for amyloid dyes consistent with the strain features observed in the respective human fAD brain [19]. Thus, mutant Aβ40 prions induce a conformation of WT Aβ similar to that found in fAD deposits, suggesting that the deposition pattern within specific regions of AD patients is likely defined by the first stable Aβ prion formed through a kinetically dominant self-propagation process, ultimately defining diverse AD phenotypes [19].

Recently, another group showed that in both sporadic and inherited forms of AD, amyloid aggregates differ in the biochemical composition of Aβ species, including aggregation kinetics, the resistance to degradation by proteases and the seeding ability [21]. As in the other studies, they also confirmed that brain homogenates from AD patients with different molecular profiles of Aβ are able to induce distinct patterns of Aβ-amyloidosis when injected into mice [21].

## 4. Tau

### 4.1. Tau Protein and Tauopathies

Tau proteins are members of the microtubule-associated proteins (MAP) family. In humans, they are found mainly in neurons [149], with trace amounts in peripheral tissues [150] and in nonneuronal cells, especially in pathological conditions [151]. Although people commonly refer to tau as a single protein, they are actually a small family of six isoforms, originating from the alternative splicing of the *MAPT* gene located on chromosome 17q21 [150] in humans. Given this clarification, from now on, we will refer to tau as a single entity wherever it is not necessary to discriminate. *MAPT* gene has 16 exons, but only some of them are constitutively translated [152]. Exons 2, 3 and 10 are alternatively spliced in the adult brain, giving rise to the different isoforms, that range from 37 to 46 kDa [153]. *MAPT* exons 2 and 3 encode each for an N-terminal insertion of 29 amino acids, and exon 3 is never present without exon 2. Exon 10 encodes the second (R2) out of four highly conserved imperfect repeated regions of 31 amino acids, which constitute the microtubule-binding domain of tau proteins, the other being encoded by exons 9, 11 and 12. Taken together, these splicing variations yield six tau isoforms that differ for the presence of zero, one or two N-terminal insertions (0N, 1N and 2N) and the presence of either 3 or 4 imperfect repeats (3R and 4R) in the C-terminal part of the protein.

Tau protein expression is developmentally regulated, with fetal brain expressing only 0N3R tau (also called “fetal tau”), while in adult CNS all isoforms are present, although at different levels [154]. The ratio between 3R- and 4R-tau isoforms is around 1:1 in healthy brains. Maintaining this equilibrium is crucial to avoid the onset of pathological conditions, as many tau-related disorders show the prevalence of one class of isoforms over the other [155]. Tau protein functions are strictly related to its structure. Like α-synuclein, tau is a hydrophilic protein which maintains a highly flexible and unfolded conformation in solution [156]. The N-terminal part has a high content of acidic residues through which it interacts with cytoskeletal components [157] and cytosolic organelles [158] and determines the spacing between microtubules, thus affecting the diameter of axons [159]. The central proline-rich sequence is involved in interactions with receptor proteins [160,161], mediating many important roles in signal transduction pathways.

The repeated regions R1–R4 located in the C-terminal part of the protein are involved in the binding of tau to the microtubules network, along with the less-conserved inter-repeat regions. In particular, they regulate the tau ability to promote the polymerization of tubulin into mature microtubules, inhibit the rate of depolymerization [162] and are involved in axonal transport [163,164,165]. Tau proteins undergo a number of posttranslational modifications, including ubiquitination, glycation, acetylation, nitration and phosphorylation (for a complete overview, see Reference [166]). More than 80 phosphorylation sites have been identified along the amino acidic sequence [152], suggesting that this modification has deep impacts on tau physiological functions. The addition of phosphate groups on specific residues of tau is essential to modulate its binding affinity for the microtubules [167]: the higher phosphate groups content, the lower the ability to interact with tubulin. Tau hyperphosphorylation on the proline-rich region [168] and the C-terminus [169] induce the self-aggregation of the cytosolic protein, which forms first oligomeric species and then insoluble fibrils. Several lines of evidence indicate that abnormal tau phosphorylation might promote neurodegeneration also by compromising axonal integrity and synaptic functions [170] and by protecting it from degradation by the proteasome system [171].

Neurodegenerative conditions known as tauopathies have as a common feature the accumulation of insoluble inclusions called neurofibrillary tangles in the cell bodies of neurons and glia. Interestingly, the pattern of deposition and the biochemical characteristics of tau aggregates are often disease-specific, suggesting the existence of different tau strains. Remarkably, tau pathology can either be the primary cause of disease or can present itself in co-occurrence with the deposition of at least another amyloidogenic protein, like α-synuclein or huntingtin.

A comparative biochemistry of tau pathological aggregates shows that they differ in both phosphorylation and content of tau isoforms, which enable a molecular classification of tauopathies. In fact, according to the biochemical properties of aggregated tau proteins, a sort of “bar code” has been established to classify tauopathies (Figure 3B) [172]. Class 0 includes rare syndromes in which there is complete loss of tau protein expression. Class I is the most populated, comprising around 10 diseases characterized by three electrophoretic bands at 60, 64 and 69 kDa and by a fourth, weaker band at 70/74 kDa. This profile corresponds to the presence of aggregates made of all six isoforms. The prototypical neurological disorder that characterizes class I is Alzheimer’s disease, but this class includes nine additional neurological disorders such as hippocampal tauopathy in cerebral aging, amyotrophic lateral sclerosis parkinsonism-dementia complex of Guam, Parkinsonism with dementia of Guadeloupe and many others. Class II includes only 4R tauopathies, in which tau amyloids form a doublet at 64 and 69 kDa. This pathological tau profile is observed in Progressive Supranuclear Palsy (PSP), Corticobasal Degeneration (CBD), Argyrophylic Grain Disease (AGD) and FTDP-17. Class III includes a single neurological disorder that is Pick’s disease (PiD). Class III tauopathies show a doublet at 60 and 64 kDa, corresponding to aggregates of 3R tau. The last class (i.e., IV) includes only specific forms of myotonic dystrophy characterized by the accumulation of aggregated fetal tau at 60 kDa.

In AD, the intraneuronal spreading of tau pathology goes together with the extracellular accumulation of Aβ plaques, which the relationship with tau-mediated toxicity is not yet clear. The main components of AD tangles are paired helical and straight filaments containing both 3R and 4R isoforms with a high degree of phosphorylation. The one-to-one ratio of 3R and 4R tau in the deposits is a peculiar characteristic of only some tauopathies, like AD and Frontotemporal Dementia linked to chromosome 17 (FTLD-17), while the other syndromes all show a dramatic prevalence of one class of isoforms [172]. In PSP, AGD and CBD, the aggregates are made exclusively of 4R tau, while Pick’s Disease is a 3R-tauopathy. The presence of different isoforms reflects distinct electrophoretic profiles from diseased brains, as well as in filaments assembly. A cryo-EM and immune-EM analysis of both PHF and SF from multiple cases of sporadic and inherited AD showed that the core structures of all the filaments are well-conserved and adopt a common fold [173]. Tau fibrils from Pick’s disease adopt instead a different conformation, which shares a similar pattern with AD but results in a distinct overall beta packing [174]. These findings establish the existence of molecular conformers of tau filaments and suggest that additional folds may be found in other tauopathies, such as PSP and CBD, which share the same isoform composition. Such diversity, mainly at turn residues between conserved secondary structure motifs as in PiD and AD filaments, might be responsible for the unique phenotypes of different 4R tauopathies.

### 4.2. Prion-Like Properties of Tau

As for other proteins mentioned in this review, the tau protein also appears to share some key features with the prion protein. In 2009, Clavaguera et al. showed that brain extracts from tau P301S-expressing mice injected into wild-type hosts triggers wild-type tau assembly into filaments and the spreading of the pathology to the regions surrounding the injection site [175]. This work highlighted another important feature of aggregated tau, that is, the ability to template the misfolding of the endogenous protein and its assembly into fibrils. An in vitro study went deeper into this issue, identifying aggregates composed at least of three protein monomers as the minimal propagation unit necessary for the internalization and induction of seeding in a HEK293 cell line [176]. Specifically, the aggregation-competent portion of the tau protein must include the microtubule binding domains, as the formation of fibrils is strictly dependent on the presence of at least one of the two hexapeptide motifs, ^275^VQIINK^280^ and ^306^VQIVYK^311^, located respectively at the beginning of the third (R3) and of the second (R2) repeated regions [177]. A subsequent number of studies conducted both in cell and animal models further defined the prion-like properties of aggregated tau protein. In these works, tau seeds were collected either from patients with an array of tauopathies [178] or from symptomatic transgenic mice [179] or generated by in vitro fibrillization reactions from recombinant material [18,180]. In all cases, amyloid tau recruited the native protein and triggered its aggregation, although with variable seeding potency.

The observation that tau aggregates derived from diseased brain homogenates have a 10-fold higher seeding activity compared to synthetic amyloids and that recombinant monomeric protein acquires the molecular properties of the in vivo aggregates if seeded with in vivo produced amyloids [179] suggests that conformational differences deriving from the aggregation process may account for the variations in seeding capacity. This has been shown also in other works where distinct conformers of tau were able to faithfully replicate their own morphologies in the newly formed aggregates, both in vitro [18] and in vivo [178,181]. Furthermore, tau-induced pathology does not remain limited to the injection site but propagates to the neighboring regions, following synaptic connections. It is now widely accepted that tau aggregates move transsynaptically and are taken up by the cells through multiple mechanisms, including micropinocytosis [179], heparan sulphate proteoglycans [36,182], bulk endocytosis [183] and tunneling nanotubes [184,185]. In the study mentioned above [175], Clavaguera et al. showed also the spreading of tau pathology from the transenthorinal cortex of mice to the hippocampus and cerebral cortex, which are anatomically connected to the injection site. Others confirmed this pattern of propagation [180,186,187], finding aggregated tau also in distant brain areas like the olfactory and limbic systems [187]. The appearance of pathological tau species in brain areas around the injection site is often accompanied by the selective loss of neurons in those regions [188]. Using a model in which tau transgene expression was restricted specifically to the enthorinal cortex, de Calignon [189] et al. and Liu et al. [190] unequivocally proved the hypothesis of the transsynaptic spreading of tau aggregates, as they were able to observe tau pathology in regions not expressing the human transgene but anatomically connected to the enthorinal cortex. Furthermore, mice with impairments in exosomal transport and in microglia, which plays key roles in phagocytosis and exocytosis, show a drastic reduction in tau pathology spreading [191]. Altogether, these data support the idea of tau acting as a prion-like protein; however, the parallelism with the prion protein does not apply when considering prion infectivity. Indeed, to date, no cases of transmission of tauopathies between individuals have been reported.

### 4.3. Tau Strains

The morphology of tau assemblies could be an important determinant in defining the type of disease that will develop. Indeed, if we consider the three classes of tauopathies (3R, 4R and 3R/4R), we could immediately notice that the protein material is the same, but it must be assembled in specific conformations that end up causing distinct pathologies. Structural insights into filaments made of recombinant tau isoforms proved that at least three types of fibrils exist, each one with individual characteristics: 3R aggregates, 4R aggregates and mixed aggregates composed of both types of isoforms that co-assemble into heterogeneous amyloids [192]. Thanks to their conformational plasticity, tau isoforms assume multiple conformations when grown on homogeneous or heterogeneous fibrils. However, an asymmetric seeding barrier exists between the 3R and 4R isoforms, probably dictated by the secondary structures that each type of isoforms assumes. While 3R tau can recruit all isoforms, 4R tau is not able to interact with 3R tau, and when they are both present, each one forms its own single fibers, which then combine into the mixed aggregates found in AD [193,194]. A high-resolution of AD-containing filaments were recently obtained from material purified from AD brains, confirming that the number of R regions deeply impacts fibrils formation and morphology [195].

Mutations in the primary sequence of tau are responsible for the vast majority of tau pathologies, and currently, more than 32 mutations have been identified in over 100 families [196]. Half of these mutations impact at the protein level, altering tau interaction with microtubules and promoting its assembly into abnormal filaments. The different biochemical properties of the mutated amino acid might also affect the hydrophobic and long-range interactions that allow fibrils formation, therefore affecting the final conformations of the assemblies. Indeed, using circular dichroism and spectroscopy techniques, Frost et al., showed that aggregates composed of wild-type or mutated (P301L/V337M) tau have different secondary structures and morphology [197]. In particular, the latter displays a curved morphology and lacks the typical helical appearance of the wild-type fibrils. A similar effect on the overall structure of the aggregates has been observed also for other amino acidic substitutions located in the central and C-terminal part of the sequence [198], as well as a different sensitivity to proteolytic cleavage [199]. Strikingly, each specific conformer could template the misfolding of the monomeric protein carrying the same mutation but was less efficient in seeding all the others, thus pointing towards a strain-like behavior of the aggregates. However, in some cases the copresence of two or more different tau proteins triggers cross-seeding events and gives rise to brand new morphomers, which, in turn, propagate their structure in subsequent passages. While P301L was not able to misfold wild-type tau after one single round of seeding reaction [199], when the double mutant P301L/V337M was used, a new conformer, termed WT*, was produced after four rounds of seeding reaction of the wild-type monomer [197].

It is noteworthy that although the protein composition of WT* fibrils was the same of the wild-type aggregates, their tertiary structure was clearly distinct, with a greater content in random coil elements and a reduced number of alpha helices. Indeed, electron microscopy analysis revealed a stronger similarity with mutated fibrils, which had been used as a template only in the first cycle of the seeding reaction. This peculiar conformer can be classified as a proper strain, since it is able to maintain its structural characteristics over time. The ability to form stable strains is not exclusively dependent on the presence of mutations, although the molecular basis of the phenomenon remains largely unknown. The exposure of cells expressing only the microtubule-binding domains of tau fused to YFP (HEK293-RD-YFP) to synthetic fibrils led to the obtainment of 20 distinct clones, among which the most representative (clone 9 and clone 10) were selected and further characterized [18]. Differences were clearly visible in the inclusion morphology, aggregate size and subcellular localization and extended also to biochemical properties, like protease sensitivity, seeding capacity and toxicity. Moreover, all the specific characteristics are maintained when the aggregates are transferred to naïve cells, and also to transgenic P301S mice where they initiate unique pathological phenotypes in distinct cell types. The authors propose that clone 9 and clone 10 might represent the final products of specific cell responses to different aggregates morphologies, mirrored by their cell compartmentalization. The pattern of spreading followed by clone 9 in mice gave further prove of the ability of tau aggregates to move through anatomic connections.

Guo et al. [200] identified striking differences in the morphological structure of AD-seeded fibrils compared to in vitro ones, which affected their seeding potency and deposition pattern. This phenomenon is not limited to AD; indeed, the inoculation of mice transgenic for human wild-type tau (ALZ17) with brain extracts from patients with different tauopathies recapitulated the major features of each disease [178]. After 6 months from the injection, all injected mice developed inclusions in the injection site, and in some cases (brain extracts of AGD, PSP and CBD), the morphologies of the newly formed aggregates were specific and consistent with the human counterparts in the brains of patients. It, thus, appears that several tau conformers made of the same tau isoform (4R tau in this case) exist in different tauopathies. Aggregates from Pick’s Disease were less efficient in templating the misfolding and aggregation processes, probably due to the lower tendency of 3R tau to assemble into amyloid structures. However, using the ultrasensitive technique, Real Time Quaking Induced Conversion reaction (RT-QuIC), Saijo et al. [201] were able to detect the seeding activity in PiD brain extracts even at very high dilutions. The same cell model that allowed the identification and characterization of the two strains termed clone 9 and 10 was then applied to further investigate the contribution of strains to the spectrum of human tauopathies [18]. Twenty-nine samples from patients with a variety of tauopathies served as seeds to template the aggregation of tau RD-YFP, which formed several inclusions that were subsequently characterized and scored according to their morphology. The analysis revealed distinct strain compositions across the pathologies, with AD showing the highest homogeneity probably correlated to the existence of a predominant strain. On the contrary, other diseases had strong interpatient variations, with at least two individual strains identified (PSP and AGD). Notably, the homogeneous behavior of AD-induced cellular aggregates mirrors the in vivo pathology, which is characteristically more uniform.

Although these data represent a substantial achievement in the overall understanding of the strain phenomenon, the authors themselves point out that other different conformers could be present that did not succeed in seeding tau RD-YFP and, thus, could not be detected. Moreover, some strains from the PiD and AGD samples were not stable in cell culture, impeding their further characterization. To test whether these putative strains were able to reproduce their array of pathological phenotypes also in vivo, mice expressing human 1N4R tau P301S were inoculated with cell lysates from each line used to amplify the strains [181]. Regarding the rates of propagation throughout the brain, in vivo data correlated with that observed in vitro, with the strains with higher seeding activity spreading faster, except for strain 10 which remained confined in the contralateral region of the hippocampus. All injected mice showed signs of tau pathology, albeit with great variations only in part consistent with the in vitro behavior. While the amount of AT8-positive aggregates paralleled with the seeding activity of the strain, the morphologies of the aggregates were unique for the specific strain, ranging from typical grain pathology (DS18) to fibers “wisps” resembling neuropil threads (DS7). Interestingly, conformers with the same proteolysis pattern also shared similar seeding activity and toxicity and induced similar phenotypes in cultured cells, suggesting that these properties are conformation-specific. Furthermore, they do not depend on the monomeric protein used as a substrate but are totally dependent on the pathological aggregate used as seed.

When brain extracts from patients with 4R and mixed tauopathies (AD, PSP and CBD) were inoculated in non-transgenic mice [202], a similar situation to P301S mice was observed, with specific inclusions forming in different subcellular localization according to the type of biological sample. AD-tau induced a thread-like pathology mainly in axons and triggered the aggregation of both 3R and 4R endogenous tau, while CBD seeds induced frequent perykarial pathology made predominantly of 4R tau. Among PSP strains, one in particular that came from the frontal cortex of the patient was found to be uniquely aggressive, with a very fast rate of spreading throughout the brain. Although showing variable seeding potency, all the three groups of strains maintained cell specificity for neurons or glia as in the corresponding disease such that only PSP and CBD samples induced astroglial and oligodendroglial pathology with coiled bodies that closely resemble their human counterparts. The presence of glial inclusions is particularly relevant, since it was believed that only neurons expressed a significant amount of tau protein. Moreover, glial amyloids significantly contributed to the spreading of the disease, either by transmission between glial cells or through the axons of neurons from the white matter. The use of non-transgenic mice as a model allowed the limitations caused by the spatial distribution of tau transgene overexpression that could influence the spreading of the aggregates to be overcome. Furthermore, the PSP and CBD strains analyzed are the first that showed consistent glial pathology.

## 5. Alpha-Synuclein

### 5.1. Alpha-Synuclein and Alpha-Synucleinopathies

Alpha-synuclein is a 140-amino-acid protein expressed mainly at the synaptic terminals of neurons in the central, peripheral and enteric nervous systems. Alpha-synuclein plays key roles in regulating the cell-to-cell communication and neurotransmitter release [203]. The protein amino acidic sequence can be divided in three segments according to their biochemical properties: the N-terminal lipid-binding domain, the amyloidogenic central portion or Non Aβ Component (NAC) and the C-terminal acidic tail. Understanding α-synuclein behavior in solution and its tendency to aggregate is crucial. The first domain spans amino acids 1–87 and is enriched in positively charged residues, organized in seven repeats of 11 aa, each containing the hexapeptidic motif KTKEGV. The amphipatic nature of these repeats induces the formation of helical structures that mediate the interaction with lipid bilayers [204]. The central core of α-synuclein (61–95) partially overlaps with the previous domain and is responsible for amyloid formation, as it forms cross-beta structures independently of the presence of the other two domains [205]. At the C-terminus, the last 43 amino acids are arranged in a random coil structure characterized by a high net negative charge and a subsequent low hydrophobicity. Intriguingly, convincing evidence proves that this domain inhibits α-synuclein aggregation by shielding the fragment of the NAC region through a long-range interaction with both the hydrophobic cluster of the NAC itself and the positive charges of the N-terminus [206]. Indeed, the C-terminal truncation of α-synuclein is enhanced in familial cases of PD and is associated with the initiation of its aggregation in vivo [207].

In addition, the C-terminal domain is homologous with the α-crystalline domain of small heath shock proteins, which are known to bind partially unfolded proteins and to prevent their accumulation into larger aggregates [208]. This similarity, together with the stability of α-synuclein in extreme conditions (high temperature and high concentration of electrolytes) points towards an involvement in the protection against thermal and oxidative stress. The native disordered conformation of α-synuclein is thermodynamically metastable [209], which means that its self-aggregation into various amyloid structures in vivo is a favorable process, tightly regulated by the proteome homeostasis and chaperone machineries. As aging progresses, the gradual impairment of the homeostatic system impairs the surveillance over the formation of aggregated species and leads to a dramatic increase in the incidence of synucleinopathies [210]. Despite being all defined by the abnormal aggregation of the same protein, α-synucleinopathies show a variety of symptoms and clinical manifestations [211]. Among them, the most studied are idiopathic PD, Dementia with Lewy Bodies (DLB) and Multiple System Atrophy (MSA). While they are all characterized by a progressive decline in motor and cognitive functions, the pattern of the lesions is specific for each disease. PD is clinically associated with motor symptoms, including rest tremor, rigidity, bradykinesia and stooping posture. Non-motor manifestations encompass neurobehavioral disorders (depression and anxiety), cognitive impairment (dementia) and autonomic dysfunction (e.g., orthostasis and hyperhidrosis). In PD, aggregated α-synuclein accumulates in neuronal perikarya (Lewy bodies) and neuronal processes (Lewy neurites). The disease process is multifocal and involves central nervous system neurons and also the enteric and autonomous nervous systems [212,213]. DLB is clinically dominated by a cognitive decline which usually precedes or begins within a year with parkinsonism. Usually dementia is associated with recurrent visual hallucinations, fluctuating cognition, rapid eye movement sleep behavior disorder and severe sensitivity to antipsychotic medications. In DLB α-synuclein aggregates present the same characteristics of those in PD, usually presenting with a major involvement of cortical brain regions [214]. MSA is defined by the presence of filamentous α-synuclein inclusions (Papp–Lantos bodies) within the cytoplasm of glial cells, together with tau- and ubiquitin-positive inclusions [215]. The symptoms include autonomic failure, urogenital dysfunctions, cerebellar ataxia and parkinsonism.

### 5.2. Alpha-Synuclein Prion-Like Properties

The first evidence for a prion-like mechanism of the α-synuclein spreading was provided by a postmortem observation of healthy neurons grafted in the brain of PD patients, which developed aggregates of α-synuclein similar to those of the host neurons [216,217]. Following these observations, Desplats and collaborators showed that α-synuclein is transmitted via endocytosis from neuronal cells overexpressing the protein to neighboring neurons, forming Lewy Bodies (LB)-like inclusions [218]. Additionally, in vivo studies showed that α-synuclein was transmitted from the affected neurons to engrafted neuronal precursor cells in a Tg mice model of PD-like pathology, leading to inclusion body formation [218]. Furthermore, MSA-derived α-synuclein aggregates inoculated in Tg mice expressing mutant α-synuclein showed the ability to recruit the endogenously expressed mutant protein and to template the formation of LBs-like structures [219]. In the attempt to confirm that the ability to induce pathology was dependent on the protein itself and was not related to unknown host factors present in the aggregates extracted from diseased brains, the efficiency of synthetic α-synuclein assemblies to induce pathology both in vitro and in vivo has been tested, demonstrating that in vitro-formed aggregates, both oligomers and fibrils, may be taken up and may propagate among cells in a prion-like manner, inducing LB-like pathology [33,218,220,221,222,223,224,225]. Indeed, human recombinant α-synuclein aggregates were able to induce the aggregation of endogenous α-synuclein in non-transfected human neuroblastoma SH-SY5Y cells [226].

Furthermore, Luk and collaborators showed that the addition of in vitro-preformed α-synuclein fibrils into a cell culture medium induces intracellular α-synuclein aggregation in different cell lines overexpressing the protein [222]. When tested in vivo, recombinant α-synuclein aggregates efficiently replicated and spread from the site of injection to anatomically connected regions in transgenic [221] and, importantly, wild-type animals [220]. Another group confirmed these results [223], showing that α-synuclein pathology caused the progressive loss of dopamine neurons in the *substantia nigra pars compacta* and caused impairment in motor coordination in mice injected with both pathological DLB brain-derived α-synuclein aggregates (i.e., the sarkosyl-insoluble fraction) and recombinant α-synuclein assemblies obtained in vitro. Alpha-synuclein pathology was absent when mice were injected with the soluble recombinant protein [223]. Subsequently, other groups showed the “infectious” activity of LB extracts from PD diseased brains in mice and also in monkeys [227]. Although all these studies clearly showed the prion properties of ɑ-synuclein assemblies [228], so far there is no evidence of pathologic α-synuclein aggregate transmission between individuals leading to the use of the prion-like definition to make a distinction between this protein and actively infectious prions [229,230].

### 5.3. Alpha-Synuclein Strains

As already mentioned, numerous studies unequivocally proved that recombinant α-synuclein aggregated in vitro can spread from cell to cell both in cultured cells [218,226,231] and in murine brains [220,221], resulting in neuronal dysfunction. Moreover, once injected, synthetic amyloids recruit the endogenous protein and template its misfolding into LB/Lewy Neurite-like structures, which closely resemble those found in patients [220,221,225]. Given the ability of α-synuclein to populate multiple conformational states in solution, changes in the environment, even minimal, can shift the equilibrium towards a specific intermediate that would grow into a distinct fibrillary assembly. Recombinant α-synuclein readily polymerizes in vitro in the presence of physiological concentrations of salts to form cylindrical structures, while under lower salt concentrations, the formed amyloids flatten and twist, resembling a sort of ribbon [232]. The different architecture of the morphomers reflects in their functional properties, as fibrils are resistant to proteinase K and exhibit a higher toxicity in cultured cells. Upon incubation with neuroblastoma cell lines, both aggregates transmit their intrinsic structures and features to endogenous α-synuclein, imprinting a unique and heritable pattern of *puncta* [232]. Although the NAC region alone is sufficient for α-synuclein fibrillization [205], the presence of the N-terminal domain and, to a lesser extent, of the C-terminus affects the final conformation of the aggregates [26]. In a work by Guo et al., full-length α-synuclein originates specific morphological aggregates, called “strain A” [26], which cannot be replicated by N-terminal-truncated forms of the protein (58–140). Self-seeding fibrillization studies showed that strain A conformation could be transmitted to the partially truncated 32–140 form of α-synuclein but not to 58–140 α-synuclein, suggesting that the segment 32–57 is essential for the strain propagation, while 1–31 is not an integral part of the core of the fibrils.

As already mentioned above, the C-terminus tends to impede α-synuclein aggregation by masking the NAC region through its negative charges. Indeed, when subjected to in vitro fibrillization, truncated 1–120 α-synuclein gives rise to a number of different conformers that cannot be stably propagated via repetitive self-seeding. This effect is in part abolished by the addition after residue 120 of a myc tag, which the negative charges of mimics in part the presence of the acidic tail and allows the formation of a different conformer, termed “strain B” [26]. Therefore, the C-terminal part seems to counteract the action of the N-terminus by impeding structural diversity. In the specific case of strain A and B, the two amyloids show a peculiar behavior in seeding both α-synuclein and tau pathology in in vivo models, with strain B being far more competent in seeding tau aggregation in transgenic mice than strain A [26]. The different inductions of tau inclusions were analyzed with a panel of antibodies that recognize pathological tau conformations. Strain-specific conformations can be imprinted also by posttranslational modifications, such as the phosphorylation of serine 129, which is a hallmark of pathological lesions in diseased brains [233]. pSer129α-synuclein fibers show a higher toxicity and distinct morphology compared to wild-type fibers and could propagate their intrinsic properties to the endogenous protein in cell cultures [234]. Interestingly, the phosphorylation of other residues did not lead to any change in fibrils morphology, implying that residue 129 must be involved in some sort of transient interaction that inhibits aggregation.

As mentioned above, the inoculation of both transgenic and non-transgenic mice with synthetic α-synuclein assemblies produces a pathology that is characterized by the presence of Lewy body/Lewy neurite structures in defined brain regions. The pattern of lesions and the pathological phenotypes observed in injected rodents are dependent on the intrinsic nature of the aggregates, thus corroborating the hypothesis of the existence of α-synuclein strains in vivo. Indeed, α-synuclein fibrils and ribbons, after mice administration, imposed a different burden on the affected brain, with fibrils resulting in progressive motor impairment and cell death while ribbons caused a distinct histopathological phenotype characterized by Parkinson’s disease and multiple system atrophy traits [27]. These appealing results provide a tentative explanation to the variability in symptoms and affected brain areas, which could be dictated by the structural and biochemical properties of the amyloids involved. The observation that only α-synuclein ribbons could propagate into oligodendrocyte cells, a feature that is typical of MSA, suggested for the first time a possible connection between distinct α-synucleinopathies and strains [232]. The inoculation of transgenic mice expressing A53T mutant α-synuclein with brain homogenates containing MSA-derived aggregates led to the instauration of a pathological condition which could be transmitted to other groups of mice faithfully propagating its own characteristics [28,219]. The accumulation of α-synuclein was primarily neuronal, probably a consequence of the weak expression of the transgene in oligodendrocyte cells; however, the peculiarities shown by both MSA seeds and spontaneous A53T seeds in Tg83^+/+^ mice and their differences in terms of distribution and incubation time are sufficient to classify them as separate strains. This notion is reinforced by the fact that PD seeds, on the contrary, failed to induce PD pathology in the same transgenic model, while in C57BL/6 mice and in macaques initiated a Lewy body-like pathology that spread throughout the CNS [227]. A similar behavior occurs for DLB-derived aggregates, which elicited α-synuclein hyperphosphorylation and aggregation into Lewy body-like structures in wild-type mice [223].

While PD and DLB aggregates triggered the aggregation and the pathological phosphorylation of the endogenous protein, MSA seeds could not propagate in the absence of A53T α-synuclein. The different identity of MSA and PD α-synuclein strains reflects in the unique clinical presentation of these disorders and in the specific aggregate deposition in the CNS: oligodendrocyte cells throughout the neuraxis in MSA, neuronal perikarya and axons of *substantia nigra* and striatum in PD. Not only the identity of misfolded seeds but also the intracellular environment prompts significantly the generation of α-synuclein strains. As shown by Peng et al., LB aggregates injected in a mice line engineered to express α-synuclein only in oligodendrocytes are able to propagate between cells, and as they spread, they lose their biochemical characteristics and acquire glial cytoplasmic inclusion (GCI)-like features, such as a higher seeding potency. Lysates of the same cells retain the ability to convert misfolded α-synuclein, in this case, synthetic preformed fibrils, into GCI-like structures. On the contrary, GCI seeds maintain their identity after multiple passages in neurons, supporting the hypothesis that the generation of their features depends on the presence of factors specific to oligodendrocytes and absent in neuronal cells [235].

## 6. TDP-43

### 6.1. TDP-43 in ALS and FTLD

One of the main characteristics of sporadic ALS (sALS) cases is the presence of motor neuronal inclusions in the CNS of affected subjects, which stain positively for ubiquitin [4]. In 2006, two different groups discovered that the protein TDP-43 was the major component of these aggregates in the spinal cord and brains of ALS subjects and of tau-negative and ubiquitin-positive inclusions in Frontotemporal lobar degeneration (FTLD) brains [236,237]. In these deposits, TDP-43 is hyperphosphorylated, ubiquitinated and abnormally cleaved to generate C-terminal fragments (CTFs). Collectively, these NDDs are named TDP-43 proteinopathies [238]. TDP-43 is encoded by the *TARDBP* gene on chromosome 1, which encodes for a 414-amino-acid protein ubiquitously expressed in all tissues and is well-conserved in invertebrates and mammals [4]. TDP-43 contains a nuclear localization signal (NLS) and two RNA recognition motifs (RRMs) followed by a glycine-rich domain. This protein is a multifunctional DNA/RNA-binding protein involved in many cellular processes, including micro-RNA processing [239], apoptosis [240], RNA transcription, alternative splicing and mRNA stability regulation [241,242], thus suggesting its essential role in many molecular and cellular processes, confirmed by the embryo lethality of its mouse knockout model [243,244,245]. The N-terminal portion of TDP-43, including two RNA recognition motifs (RRMs), is thought to have a folded conformation, whereas the C-terminal portion is unstructured and is considered to be aggregation prone [246].

After the identification of TDP-43 as the major protein component of intraneuronal aggregates in ALS and FTLD, several pathological mutations in the *TARDBP* gene have been identified in subjects affected by familial and sporadic forms of ALS and in a restricted number of subjects with FTLD [240,247,248,249], thus confirming a direct link between this protein and pathology. It is now well-known that ALS and FTLD can clinically overlap with ALS patients displaying cognitive impairment or signs of FTD in approximately 50% and 15% of the cases, respectively [250]. This strict connection has also been confirmed by a neuropathological analysis which identified TDP-43 pathology in the majority of sALS cases (around 95%) and in 60% of FTLD cases [4]. FTLD-TDP can be classified according to the shape and distribution of the pathological TDP-43 lesions in the CNS. This classification was harmonized in 2001 [251] and recently revised with the addition of a new subtype with specific clinical and histopathological features [252]. Interestingly, every histopathological subtype (from A to E) presents a distinct WB banding pattern of the hyperphosphorylated protein extracted as the sarkosyl-insoluble fraction of brain homogenates and is also related to specific clinical features and genetic background (Figure 3C) [252].

Type A is defined by large numbers of short dystrophic neurites and crescent or oval shaped neuronal cytoplasmic inclusions (NCIs). Type A is associated with behavioral variant FTD (bvFTD) or nonfluent/agrammatic primary progressive aphasia (naPPA) and mutations in the Progranulin gene (*GRN*). Type B is associated with mild levels of NCIs in all the cortical layers and only a few short dystrophic neurites. Type B has been correlated to patients with ALS and FTD and presents a link with the *C9ORF72* expansion. Type C is characterized by numerous long dystrophic neurites, mainly in cortical layer 2 and a few NCIs. Type C has been associated with the semantic dementia variant of FTD. Type C cases are typically sporadic FTD having no association with any known disease-causing genetic mutations. Type D consists of numerous short dystrophic neurites, lentiform neuronal intranuclear inclusions and a few NCIs throughout all layers of the cortex. Type D pathology is directly associated with patients with inclusion body myopathy with early onset Paget’s disease and FTD (IBMPFTD) caused by valosin-containing protein (VCP) mutations. Type E is defined by the predominance of ubiquitin-negative granulofilamentous neuronal inclusions (GFNI’s) over compact neuronal inclusions, the presence of abundant, very fine grey matter grains together with the presence of oligodendroglial inclusions. Type E is mainly associated with bvFTD, characterized by a rapidly progressive clinical course and seems to have no association with any known genetic mutation [252].

### 6.2. The Prion-Like Properties of TDP-43

Several studies have shown the intrinsic propensity of TDP-43 to aggregate [253,254,255]. The ability of TDP-43 aggregates to act as seeds in vitro and, very recently, in vivo has been demonstrated [256]. The first evidence of TDP-43 prion-like properties comes from the work of Johnson et al. in 2009 [255], which showed an increase in the turbidity of a solution containing the full-length TDP-43 recombinant protein (recTDP-43^FL^) at room temperature and constant agitation. The same experiment was repeated using the N- and C-terminal fragments of the protein, and the increase in turbidity of the solution (i.e., aggregation) was observed only in the presence of the C-terminal region of TDP-43, confirming the crucial role of this region of the protein for its aggregation. The connection between the C-terminal part of the protein and pathology has also been confirmed by a genetic analysis of the *TARDBP* gene, since the majority of the identified pathogenic mutations clusters in the region of the gene which encodes for the C-terminus of TDP-43 [240,247,248,249]. When probed with ThT or Congo red, the final products of this reaction did not show a specific binding with these dyes, suggesting the absence of β-sheet structures in recTDP-43 aggregates [255]. Although initial pathological studies suggested that TDP-43 pathological aggregates displayed nonamyloid structures [257,258], a subsequent analysis showed the presence of amyloid structures in the spinal cord of a subset of ALS patients [259] and that the use of a different pretreatment of CNS tissues form ALS and FTLD-TDP patients allowed an increased detection of amyloid positive TDP-43 inclusions [260]. Another study by Furukawa et al. showed that recTDP-43^FL^ aggregates obtained in vitro were moderately able to bind ThT, thus demonstrating the presence in their conformation of β-sheet structures [253]. When analyzed in electron microscopy, these aggregates presented a fibrillary morphology, confirming what was observed with the ThT binding. They also showed the seeding capacity of recombinant wild-type and mutant-insoluble TDP-43 aggregates into HEK293 cells under TDP-43 (with a C-terminal His-tag; TDP-43-HA) overexpressing conditions. These aggregates were able to seed the fibrillization of endogenous TDP-43-HA and contained the pathological C-terminal fragments (CTFs) within the aggregates, even if, in this case, there was no evidence of hyperphosphorylation of the aggregated intracellular protein as it occurs in ALS and FTLD-TDP diseased brains [253].

A crucial study for the demonstration of the seeding ability of TDP-43 aggregates was performed in 2013 by Nonaka and colleagues. This group transfected human TDP-43 aggregates collected from ALS and FTLD-TDP diseased brains into SH-SY5Y cells with and without overexpressing the full-length human wild-type TDP-43. Only cells which were overexpressing the protein and that were treated with the brain extract containing the pathological TDP-43 aggregates showed the presence of bands of the hyperphosphorylated protein in a WB analysis in the sarkosyl-insoluble fraction of their lysates. They also showed that pathological aggregates were toxic to cells, probably via an impairment of the cellular proteasome system and that the seeding reaction was time-dependent (i.e., bands appeared and increased in their intensity after the third day postinfection) and “self-templating” [261]. By self-templating the authors mean that the banding pattern observed at the WB analysis of the sarkosyl-insoluble fraction of the affected cells mostly corresponded to one of the pathological proteins extracted from the brain used as a seed. The seeding activity of pathological diseased brain extracts was recently confirmed by another group in a murine motor neuron-like cell line (NSC-34) under TDP-43 overexpressing conditions [262]. Finally, Porta et al. showed that intracerebral injections of biologically active pathogenic FTLD-TDP seeds into transgenic mice expressing cytoplasmic human TDP-43 and also non-transgenic mice led to the induction of de novo TDP-43 pathology. Moreover, this group showed that TDP-43 pathology progressively spreads throughout the brain in a time-dependent manner, supposedly via the neuroanatomic connectome [256].

### 6.3. TDP-43 Strains

As for TSEs, ALS also displays different phenotypes with the opportunity to have a predominant involvement of the upper or lower motor neurons, the clinical onset possibly involving spinal or bulbar muscles and, as already mentioned, the association with cognitive impairment in a continuum with Frontotemporal dementia [263]. Frontotemporal dementia, for its part, can also present with the impairment of different cognitive abilities with predominant behavioral or language impairment [252]. Furthermore, according to neuropathological examination, FTLD-TDP, as mentioned earlier, can be classified in different histopathological subtypes according to the shape and distribution of TDP-43 positive lesions [252]. All these observations led to the speculation that different conformations or “strains” of misfolded TDP-43 might be responsible for the wide variety of different clinical ALS/FTLD phenotypes related to a specific strain-related distribution in preferential CNS areas. The hypothesis of the existence of different TDP-43 aggregated species in the CNS of FTLD-TDP subjects is corroborated by the fact that there is a specific western blot banding pattern associated with each histopathological subtype (Figure 3C), thus suggesting different biochemical properties and conformations of the pathological aggregated TDP-43. Another proof in this direction was provided by the already mentioned seeding experiment performed by Nonaka et al. using SH-SY5Y cells in which it was shown that aggregates extracted from a specific brain with a predominant histopathological subtype were able to template the same biochemical properties (i.e., the same specific western blot banding pattern) on the endogenous wild-type TDP-43 protein [261]. Shimonaka et al., used TDP-43 aggregates obtained by the in vitro fibrillization of different C-terminal TDP-43 peptides to transduce cells expressing wild-type or mutant TDP-43 protein and showed that the sarkosyl-insoluble fraction of these cell lysates contained different phosphorylated C-terminal fragments of TDP-43 and different trypsin-resistant bands. These results suggest that the templated aggregation of TDP-43 by seeding with different peptides induced various types of TDP-43 pathologies, i.e., the peptides appear to act like prion strains [264]. Finally, it was recently shown that pathological TDP-43 brain extracts were able to spread in the CNS after injection in Tg and, less efficiently, in non-Tg mice. Also in this case, it was noted that not all subtypes of TDP-43 species had the same infectious ability. Moreover, this group performed an in vitro assessment of the brain extract seeding activity, observing that FTLD-TDP-*GRN* lysates had the highest activity followed by FTLD-TDP-*C9ORFf72* and sporadic FTLD-TDP cases [256]. However, more extensive biochemical and biophysical studies are needed to show the presence of distinct pathogenic TDP-43 strains in FTLD-TDP and ALS CNS, possibly related to the presence of different TDP-43 conformers (i.e., strains) with a specific tropism for selected brain areas and characterized by a different seeding ability, resulting, ultimately, in the extreme clinical heterogeneity observed in FTLD-TDP and ALS patients.

## 7. Conclusions

Although NDDs show marked differences in terms of clinical and neuropathological features, increasing evidence suggests that they share a common pathogenic characteristic: the presence of deposits of misfolded proteins with altered physicochemical properties in the CNS [1,2]. In recent years, several reports suggest that all misfolded protein accumulated in NDDs diseased brains are able to template their misfolding onto their respective native counterparts in a process which closely resembles the prion replication cycle [10], called prion-like mechanism [2]. The prion-like features displayed by misfolded proteins involved in neurodegeneration led to relevant implications. Indeed, it seems that, as prions, each protein is able to acquire not just one misfolded conformation but several and that every possible different conformer (i.e., a strain) can result from a process of selection or adaptation (Figure 2). From a single molecule or from a pool of conformers present in the affected tissue, the process could lead to the efficient replication and accumulation of a dominant one, able to prevail among the others. In this context, it should be noted that the presence of multiple pathological misfolded protein conformers in a single patient or among different affected subjects poses to the scientific community several unanswered issues:

(i) if prion and prion-like proteins are subjected to evolution, should we also expect an increase/evolution of misfolding-related disorders? In a recent work, it has been shown that vCJD infectious agent(s) contained in soluble or insoluble fractions of human vCJD blood donors was/were able to replicate in macaques generating typical and nonconventional vCJD phenotypes, the latter characterized by the presence of PK-sensitive PrP^Sc^ deposition and atypical clinical features mainly involving the spinal cord [93]. These results clearly suggest that prions show the ability to modify not only their structure but also their clinical manifestations after the infectious passage between human donors and nonhuman primates and that this “evolution” is dependent on the infectious source utilized.
(ii) How accurate are the diagnostic tools that we are currently using to discriminate between different NDDs entities? In fact, if diagnostic molecules are not able to bind all the possible misfolded conformers, as it was shown for the PiB ligand for Aβ [24,25,148], it is not possible to reliably classify the disease. Such a lack of reliable diagnostic tests could reduce our accuracy in studying the neurodegenerative process and also the sensibility and specificity in selecting possible candidates for clinical studies and pharmacological trials.
(iii) The same concept applies for therapeutic molecules. As indicated by Rasmussen et al., the discovery of multiple Aβ conformers associated with different AD clinical entities suggests the need for the development of drugs with multiple targets or for the use of a pool of polyclonal antibodies directed against the wider range of amyloid pathological structures [20].

In light of all these considerations, it is of utmost importance to clearly understand the molecular basis leading to strain formation and propagation to develop targeted diagnostic and therapeutic strategies. Reconsidering proteinopathies as “conformational disorders” could allow the development of strain- and, thus, patient-oriented strategies in order to face the high heterogeneity displayed by neurodegenerative disorders.
